# The plasminogen binding protein PbsP is required for brain invasion by hypervirulent CC17 Group B streptococci

**DOI:** 10.1038/s41598-018-32774-8

**Published:** 2018-09-25

**Authors:** Germana Lentini, Angelina Midiri, Arnaud Firon, Roberta Galbo, Giuseppe Mancuso, Carmelo Biondo, Emanuela Mazzon, Annamaria Passantino, Letizia Romeo, Patrick Trieu-Cuot, Giuseppe Teti, Concetta Beninati

**Affiliations:** 1 Metchnikoff Laboratory, Departments of Human Pathology and Medicine, University of Messina, Messina, Italy; 2 Institut Pasteur, Unité de Biologie des Bactéries Pathogènes à Gram Positif, CNRS ERL6002, 75015 Paris, France; 3 Department of Chemical, Biological, Pharmaceutical and Environmental Sciences, University of Messina, Messina, Italy; 4 IRCCS Centro Neurolesi Bonino Pulejo, Messina, Italy; 5 Department of Veterinary Sciences, University of Messina, Messina, Italy; 6 Charybdis Vaccines Srl, Messina, Italy; 7 Scylla Biotech Srl, Messina, Italy

## Abstract

*Streptococcus agalactiae* (Group B *Streptococcus* or GBS) is a frequent cause of serious disease in newborns and adults. Epidemiological evidence indicates a strong association between GBS strains belonging to the hypervirulent CC17 clonal complex and the occurrence of meningitis in neonates. We investigate here the role of PbsP, a cell wall plasminogen binding protein, in colonization of the central nervous system by CC17 GBS. Deletion of *pbsP* selectively impaired the ability of the CC17 strain BM110 to colonize the mouse brain after intravenous challenge, despite its unchanged capacity to persist at high levels in the blood and to invade the kidneys. Moreover, immunization with a recombinant form of PbsP considerably reduced brain infection and lethality. *In vitro*, *pbsP* deletion markedly decreased plasmin-dependent transmigration of BM110 through brain microvascular endothelial cells. Although PbsP was modestly expressed in bacteria grown under standard laboratory conditions, *pbsP* expression was markedly upregulated during *in vivo* infection or upon contact with cultured brain endothelial cells. Collectively, our studies indicate that PbsP is a highly conserved Plg binding adhesin, which is functionally important for invasion of the central nervous system by the hypervirulent CC17 GBS. Moreover, this antigen is a promising candidate for inclusion in a universal GBS vaccine.

## Introduction

*Streptococcus agalactiae* (also referred to as Group B *Streptococcus* or GBS), is a Gram-positive encapsulated bacterium that is frequently found as a commensal in the human gastrointestinal and genital tracts^1,2^. In addition, GBS can cause invasive infections particularly in neonates, pregnant women and elderly adults. Neonatal disease occurring during the first 6 days of life (referred to as early-onset disease or EOD) is likely due to transmission of the bacterium from the pregnant mother to the neonate secondary to aspiration of infected amniotic fluid or vaginal secretions during labor. Most cases of EOD are characterized by pneumonia followed by septicemia^3^. Late-onset disease (LOD), occurring from age 7 days to 3 months, also consists of septicemia, but displays a higher rate of meningitis, compared with EOD^4–6^.

Of the 10 GBS capsular types, the majority of invasive neonatal diseases are associated with the serotype III^7–9^. Among this serotype, one highly homogenous clone belonging to the clonal complex (CC) 17 is isolated in the majority of LODs and is referred to as the “hypervirulent” GBS clone^7,8,10,11^. Comparative genome analyses suggest an adaptation of the CC17 clones to humans, with an enhanced ability to colonize the gastro-intestinal tract and to cause invasive diseases, especially meningitis^12,13^. Several studies have attempted to explain the mechanisms underlying brain invasion by GBS and the striking meningeal tropism of the CC17 clones^14^. The first step in brain invasion involves adhesion of blood-born GBS to the microvascular endothelium, which is mediated by multiple interactions between bacterial surface proteins and cognate receptors, such as fibrinogen, laminin and fibronectin present on the endothelial lining^15–22^. After adhesion, GBS are internalized by endothelial cells and transmigrate within phagosomes^23,24^. In this process, GBS exploit the physiological process of transcytosis by which endothelial cells move macromolecular cargo from the circulation into the brain interstitium within membrane-bound vescicles^25^. In addition, bacterial toxins and proteolytic or inflammatory host factors can increase the permeability of the endothelial layer, thereby further promoting transversal of the brain blood barrier (BBB) by bacterial pathogens^26–28^. The surface proteins of CC17 strains are distinct from those belonging to other clonal complexes and might confer selective advantages during colonization or invasive infections^29^. For examples, the CC17-specific HvgA and Srr2 cell-wall proteins contribute to the increased virulence and to the meningeal tropism of CC17 strains^30,31^.

Recently, the ability of GBS to hijack the host fibrinolytic system and to invade the brain by plasminogen-dependent mechanisms has attracted considerable attention. Plasminogen (Plg) is an inactive proenzyme that is present in plasma at concentrations of approximately 2 μM and can be converted to plasmin (Pln) by host activators, such as tissue type (tPA) or urokinase type (uPA) activators. Contrary to Group A *Streptococcus*, which uses streptokinase to convert Plg to plasmin (Pln), GBS does not express endogenous Plg activators. Instead, after binding to the GBS surface, Plg is converted to Pln by host tPA or uPA during infection^32,33^. Pln, in turn, contributes to a number of amplification loops leading to increased Plg activation, generation of active metalloproteases, degradation of the extracellular matrix (ECM) and severe weakening of the BBB^28,32,33^.

We recently characterized PbsP (Plasminogen binding surface Protein), a cell wall-anchored protein that is required for acquisition of surface-associated Pln activity by GBS and for its dissemination from the blood to the brain^34^. This protein is a valuable vaccine candidate since PbsP is highly conserved (>99.3% identity at the protein level) and the *pbsP* gene is present in all sequenced human GBS strains. However, expression of PbsP at the GBS cell surface was shown to be strain-dependent^34^. Immunization with PbsP is protective against infection with the CC23 NEM316 strain^34^, but the role of PbsP in the virulence and meningeal tropism of the CC17 strains is unclear. In contrast to CC23 strains, the protein is expressed at very low levels on the surface of CC17 strains grown under standard laboratory conditions^34^, which may greatly limit the potential usefulness of PbsP in an anti-GBS vaccine^34^.

The present study was undertaken to investigate whether PbsP has a role in the context of infections caused by CC17 GBS and whether immunization with PbsP is effective against this major strain lineage causing meningitis in neonates. Using a mouse model of meningitis, we demonstrate upregulation of PbsP in CC17 GBS specifically during infection and its role in the invasion of the central nervous system. Accordingly, PbsP expression increases *in vitro* upon contact with cultured brain endothelial cells and PbsP has a non-redundant role in plasmin-dependent transmigration through monolayers of endothelial cells. In addition, we demonstrate that immunization with PbsP prevents the development of meningo-encephalitis in mice infected with CC17 GBS.

## Results

### PbsP is required for virulence of CC17 GBS

**Figure 1:**
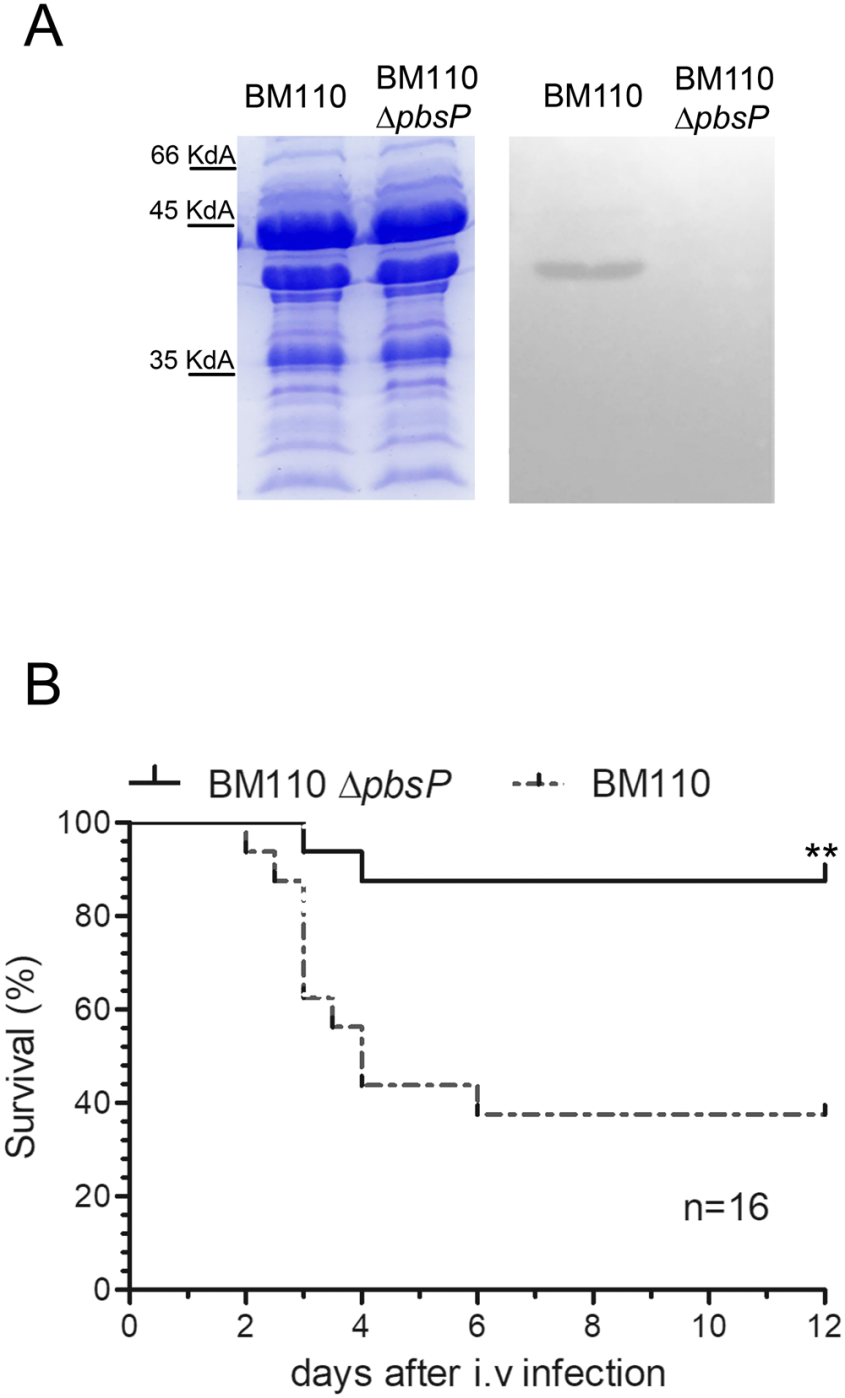
*PbsP* is required for virulence of CC17 GBS. (**A**) Western blot analysis: 10 µg of cell wall extracts from the wild-type CC17 BM110 strain (BM110) and its *pbsP* deletion mutant (BM110 Δ*pbsP*) were run on SDS-PAGE gels. Duplicate gels were stained with Coomassie (left) or blotted on nitrocellulose membranes for western blot analysis (right) using a polyclonal anti-PbsP mouse serum followed by peroxidase-conjugated goat anti-mouse IgG. (**B**) Effect of *pbsP* deletion on survival of BM110-infected mice. Adult CD1 mice were infected intravenously (i.v.) with 1 × 10^8^ CFU of wild-type BM110 or its *pbsP* deletion mutant (BM110 Δ*pbsP*). Survival was monitored every 12 h. **p < 0.01 by Kaplan-Meier analysis. Shown are the cumulative results of two experiments, each involving 8 mice per group.

To investigate the role of PbsP in the pathogenesis of invasive GBS infection, the *pbsP* gene was deleted in-frame in the chromosome of the CC17 strain BM110. The viability, morphology and growth in Todd-Hewitt broth (THB) of the ∆*pbsP* mutant were similar to those of the wild type (WT) parental strain (Figs S1 and S2). Western-blot analysis of GBS cell wall extracts using a polyclonal mouse serum (pAb) raised against recombinant PbsP showed a reactive band in the WT strain that was absent in the ∆*pbsP* mutant (Fig. 1A). We next compared the virulence of BM110 WT with that of the mutant using a mouse model of invasive GBS infection involving hematogenous spreading of the bacteria to the brain after intravenous challenge. Under these conditions, survival was increased in mice infected with the ∆*pbsP* mutant and, at the end of the experiment, only 2 out of 16 (12%) of the mice infected with the mutant strain died, while 10 out of 16 (62%) of those challenged with the WT strain succumbed to infection (Fig. 1B).

**Figure 2:**
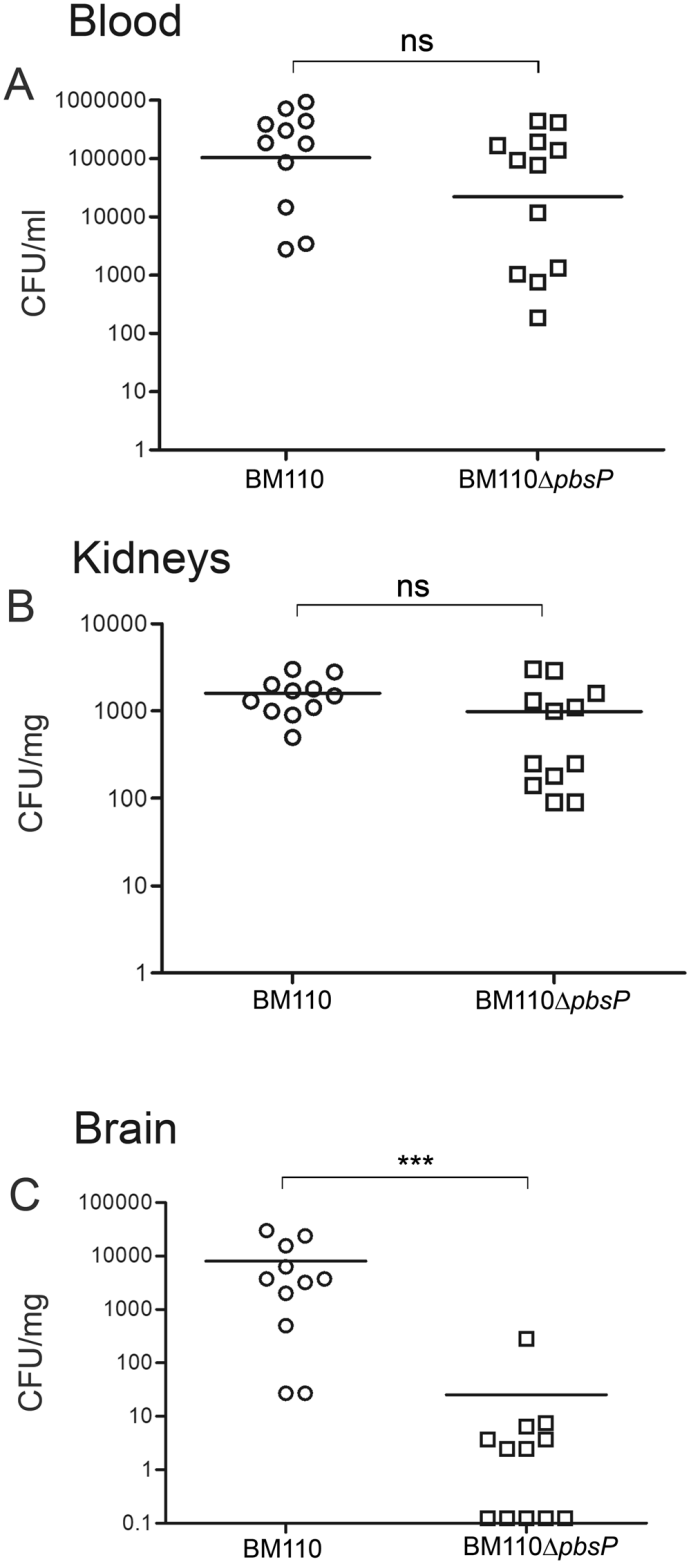
*PbsP* is required for brain invasion by CC17 GBS. Bacterial burden in the blood (**A**) kidneys (**B**) and brains (**C**) at 48 h after i.v. challenge with 1 × 10^8^ CFU of wild-type BM110 GBS (BM110) or its *pbsP* deletion mutant (BM110 Δ*pbsP*). ***p < 0.001 by the Mann-Whitney *U* test; ns, non-significant. Shown are cumulative results from 2 experiments, each involving 6 mice per group.

To determine whether the decreased lethality induced by the ∆*pbsP* mutant was linked to an impaired ability to invade the central nervous system, we compared the ability of BM110 WT and ∆*pbsP* mutant to persist in the blood and to colonize the kidneys and brains at 48 h after infection. Mice challenged with the WT strain had significantly higher bacterial counts than those infected with the Δ*pbsP* mutant in the brain, but not in the blood or kidneys (Fig. 2A–C).

**Figure 3:**
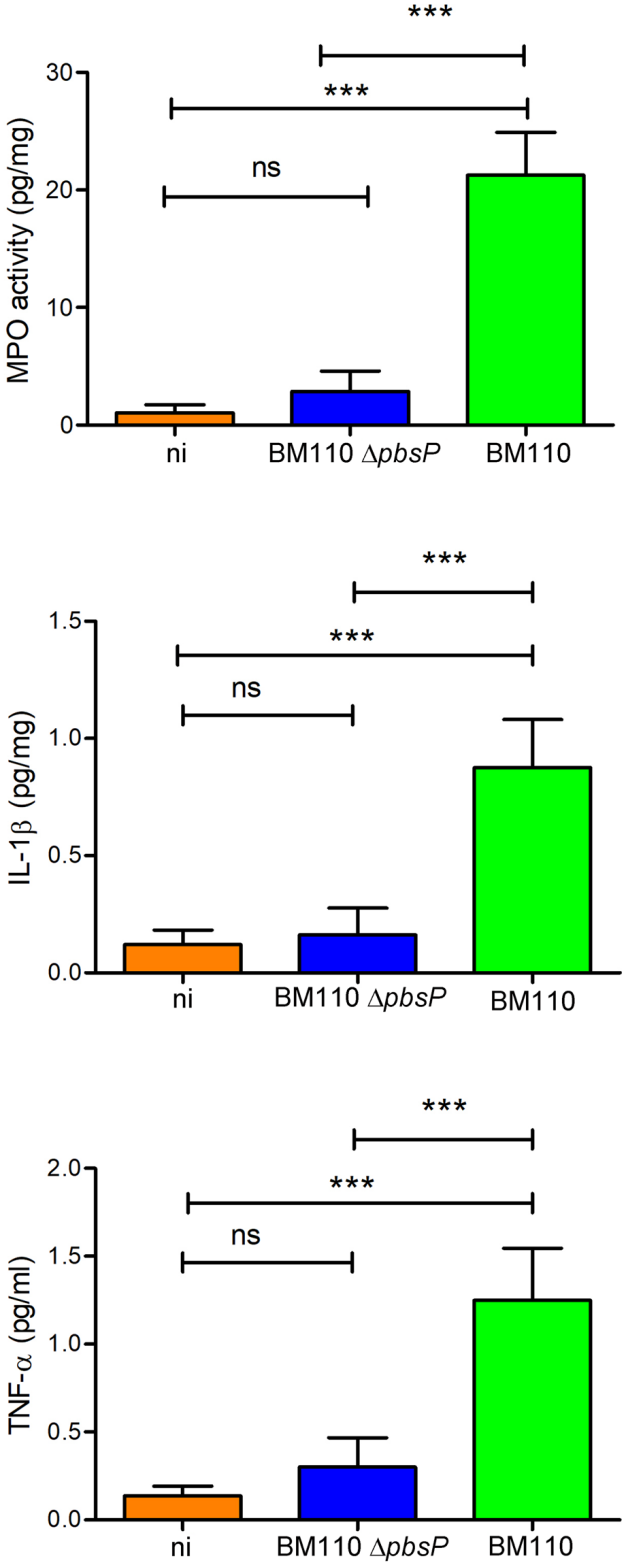
*PbsP* is required for the development of GBS-induced encephalitis. Myeloperoxidase (MPO), interleukin beta (IL-1β) and tumor necrosis factor-alpha (TNF-α) protein levels were measured in organ homogenates from wild-type BM110 GBS (BM110) or its *pbsP* deletion mutant (BM110 Δ*pbsP*). ni, non-infected. Data are expressed as means ± standard deviations of three observations, each conducted with a different animal. ***p < 0.001 by one-way ANOVA; ns, non-significant.

Next, we determined whether infection with the Δ*pbsP* mutant was associated with decreased brain inflammation, which is a major determinant of lethality in this animal model. To this end, we measured the levels of pro-inflammatory cytokines and of myeloperoxidase (MPO), an indicator of neutrophil infiltration, in brain homogenates of infected mice. MPO, interleukin-1 beta (IL-1β) and tumor necrosis factor-alpha (TNF-α) levels in the brains of mice infected with the Δ*pbsP* mutant were not significantly different from those measured in uninfected mice and were markedly lower than those measured in mice infected with WT BM110 (p < 0.001; Fig. 3). Therefore, these data indicate that PbsP selectively promotes hematogenous spreading of bacteria to the brain and the subsequent development of encephalitis.

**Figure 4:**
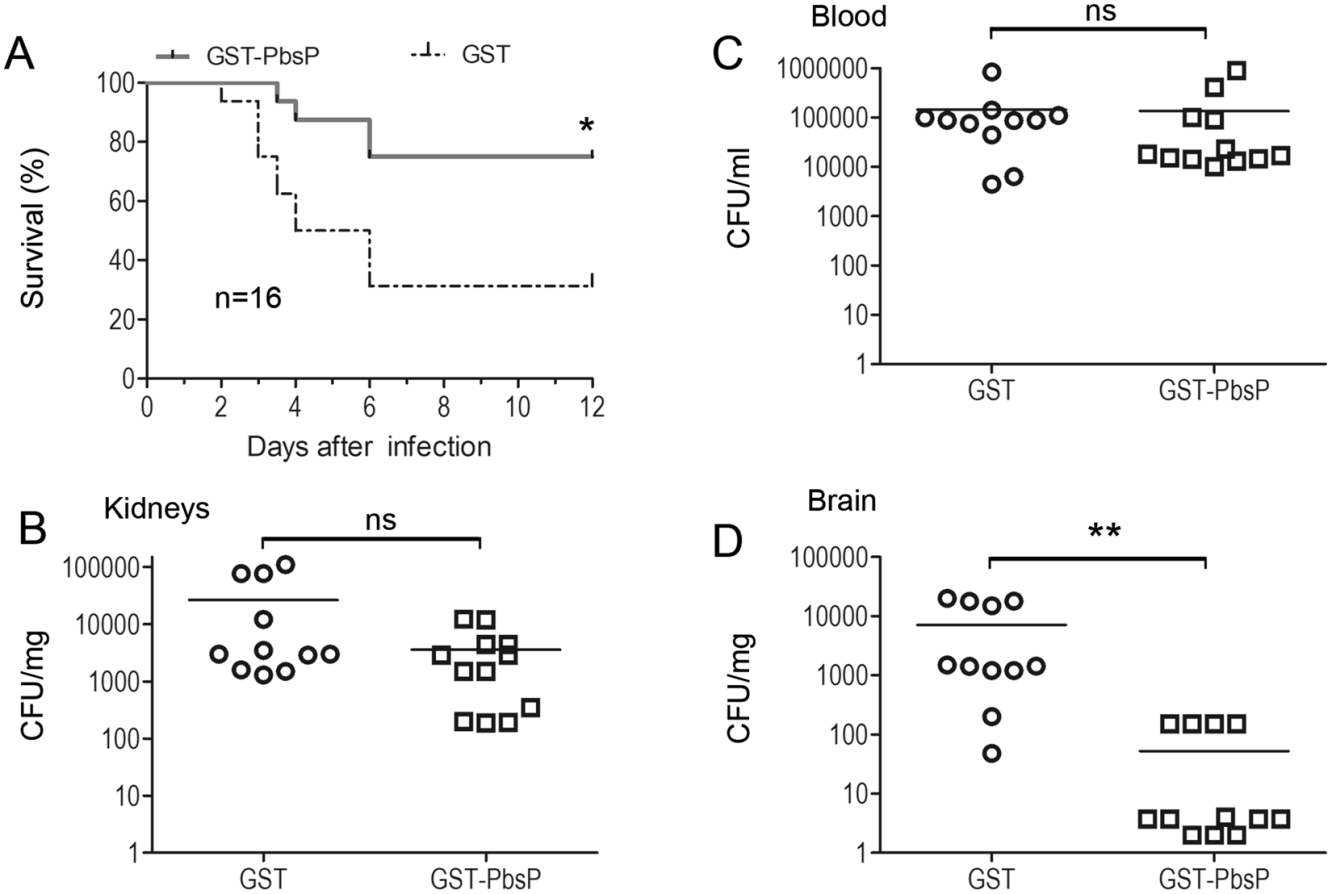
*PbsP*-based immunization protects against lethal CC17 GBS infection in mice. (**A**) Mice were immunized with recombinant PbsP fused to glutathione-S-transferase (GST-PbsP) or with GST as a control and challenged i.v. with 1 × 10^8^ CFU of wild-type CC17 strain BM110. *p < 0.05 by Kaplan-Meier analysis. Shown are the cumulative results of two experiments, each involving 8 mice per group. (**B**–**D**) Effects of PbsP immunization on organ bacterial burden after i.v. challenge with 1 × 10^8^ CFU of BM110. **p < 0.01 by the Mann-Whitney *U* test; ns, non-significant. Shown are cumulative results from 2 experiments, each involving 6 mice per group.

Since this antigen is an interesting vaccine candidate due to its high degree of conservation among GBS isolates^34^, we next investigated if immunization with PbsP prevents invasive disease by CC17 GBS. Adult mice were immunized with GST-PbsP or GST, used as a negative control, and challenged intravenously three weeks later with WT BM110. Under these conditions, immunization with PbsP resulted in increased survival (75% versus 37% in immunized and control animals, respectively; P < 0.05 by log-rank Kaplan-Meyer analysis) and in decreased bacterial colony counts in the brain but not in the blood or kidneys at 48 h post challenge (Fig. 4). In addition, lower levels of inflammation markers were measured in brain homogenates from PbsP-immunized animals relative to those from mice immunized with the GST control protein (Fig. S3).These data indicate that PbsP immunization can induce significant protection against encephalitis and lethality produced by CC17 GBS.

### PbsP is highly expressed *in vivo*

**Figure 5:**
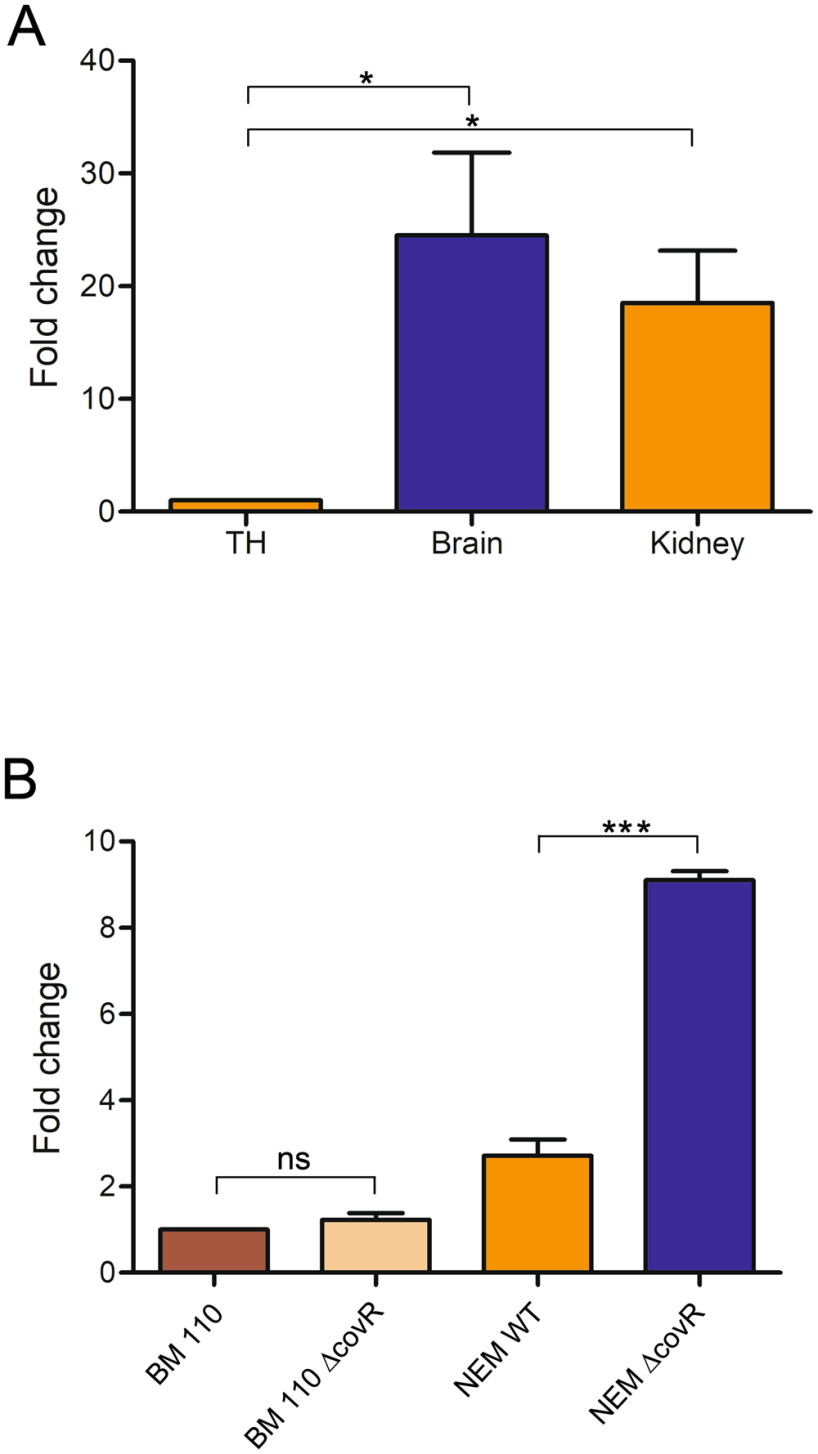
Expression of *pbsP* is upregulated *in vivo*. (**A**) RT-PCR analysis of *pbsP* mRNA levels in bacteria grown in Todd-Hewitt broth (TH) or isolated from the kidneys and brains of BM110-infected mice. Values are presented as ratios relative to the values observed in bacteria grown in TH broth. Results are means ± SD from four independent experiments performed in triplicate. (**B**) RT-PCR analysis of *pbsP* mRNA levels in wild-type CC17 BM110 strain (BM110), wild-type CC23 NEM316 strain (NEM316) and their respective *CovR* deletion mutants (BM110 Δ*CovR* and NEM316 Δ*CovR*). Values were normalized for those observed in wild-type BM110. Results are means ± SD from three independent experiments performed in triplicate. *p < 0.05; ***p < 0.001 by Student’s t test; ns, non-significant.

The protective activity of PbsP after immunization was unexpected since this protein is expressed at low levels on the surface of CC17 GBS strains, at least following growth under standard laboratory conditions^34^. We thus investigated whether PbsP is upregulated *in vivo* by performing quantitative RT-PCR analysis of bacterial RNA extracted from the kidneys and brains of mice infected with the BM110 strain. Figure 5A shows that PbsP mRNA levels were approximately 20–30 fold higher in bacteria harvested from the organs of infected mice, as compared with those grown in Todd-Hewitt broth, indicating that *pbsP* transcription is markedly upregulated *in vivo*.

In the CC23 NEM316 strain, PbsP expression is increased in the absence of the CovRS two-component system (TCS), the master regulator of GBS virulence^34,35^. However, this was not the case for the CC17 strain BM110 where only a slight, non-significant, increase in *pbsP* mRNA levels was observed in the *∆covR* mutant compared to the WT BM110 (Fig. 5B). To ascertain whether the differential effects of CovR on *pbsP* expression in the two clonal complexes could be explained by differences in regulatory sequences, we compared the promoter regions of PbsP in NEM316 and BM110 genomes^36^. However, these sequences were identical in both strains (data not shown), suggesting that the regulatory role of CovR on *pbsP* expression in NEM316 is not direct, in agreement with our previous data showing that CovR do not bind to the promoter region of PbsP^34^.

### PbsP promotes interactions of BM110 GBS with brain endothelial cells

**Figure 6:**
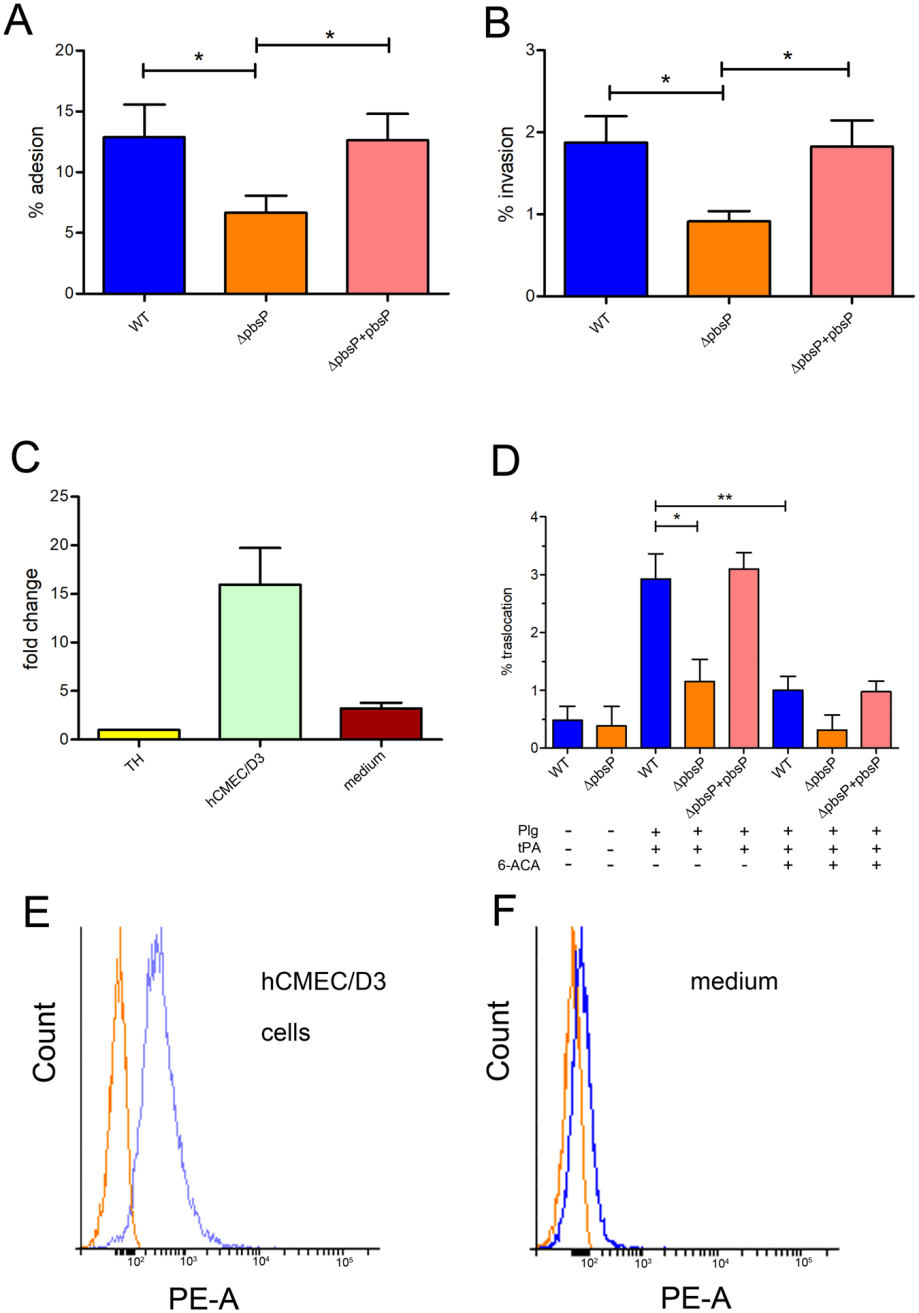
*PbsP* is required for adhesion to and transmigration across brain endothelial cells by CC17 GBS. (**A**,**B** and **D**) GBS strains were compared for their ability to interact with endothelial cells. WT, BM110 wild-type strain; Δ*pbsP*, isogenic BM110 *pbsP* deletion mutant; Δ*pbsP* + *pbsP*, Δ*pbsP* strain carrying a complementing vector with constitutive *pbsP* expression. Results are means ± SD from four independent experiments performed in triplicate. *p < 0.05 and **p < 0.01 by Bonferroni test and one way ANOVA. Adhesion (**A**) invasion (**B**) and traslocation (**D**) were assessed using the brain endothelial cell line hCMEC/D3. Plg, plasminogen; (tPA), tissue Plg activator; (6-ACA), 6-amino-n-caproic acid (100 nM). (**C**) RT-PCR analysis of *pbsP* mRNA levels in BM110 GBS incubated for 3 h in the presence of hCMEC/D3 cells (hCMEC/D3) or fresh, cell-free hCMEC/D3 culture medium (cell culture medium). Values were normalized for those observed in bacteria grown in Todd-Hewitt broth (TH). (**E**, **F**) Immunofluorescence flow cytometry analysis using mouse polyclonal anti-PbsP serum (blue line) or control anti-GST serum (red line) of PbsP surface expression in BM110 GBS exposed to cultured brain micro-vascular endothelial cells as detailed in (**C**).

Since invasion of the endothelial barrier is a critical step in brain infection by GBS^37,38^, we compared the adhesion and invasion properties of the Δ*pbsP* mutant with those of the parental BM110 strain using the brain endothelial cell line hCMEC/D3. The deletion mutant was significantly impaired in its ability to both adhere to and invade hCMEC/D3 cells compared to the WT and to the complemented strain (Fig. 6A,B). We next investigated whether PbsP is upregulated under the conditions used in the adhesion and invasion assays. We observed a 12- to 15-fold increase of *pbsP* mRNA expression in BM110 GBS incubated with hCMEC/D3 monolayers, relative to the values observed with bacteria incubated in cell-free cell culture medium (Fig. 6C). Increased expression of PbsP in bacteria exposed to hCMEC/D3 was also confirmed at the protein level by immunofluorescence flow cytometry analysis using anti-PbsP antibodies (Fig. 6E,F). These data indicate that PbsP expression is upregulated in BM110 after contact with brain vascular endothelial cells and that this surface protein is required for adhesion and invasion.

Previous studies using the CC23 NEM316 strain indicated that, after Plg binding to the bacterial surface, conversion of the zymogen to Pln by host tissue tPA is important for migration across brain endothelial cells^29,34^. Therefore, we investigated whether transmigration across the brain endothelium is Pln-dependent also in the context of CC17 strains and whether PbsP plays a role in this process. We first observed that the ability of WT CC17 BM110 to migrate through hCMEC/D3 monolayers was largely Pln-dependent, as it was markedly decreased in the absence of either Plg or tPA (Fig. 6D). We have previously shown that lysine and the lysine analog 6-aminocaproic acid (6-ACA) selectively inhibit PbsP binding to Plg, suggesting that PbsP might engage lysine binding sites on the kringle domains of the zymogen molecule^34^. Accordingly, bacterial migration through hCMEC/D3 monolayers was significantly impaired by 6-ACA (Fig. 6D). Moreover, the BM110 ∆*pbsP* mutant displayed significantly decreased Pln-dependent transmigration across the monolayers, a defect which was reversed by genetic complementation (Fig. 6D). Collectively, these data indicate that PbsP plays a crucial role in Pln-dependent transmigration of the CC17 strain BM110 across brain endothelial cells.

## Discussion

The present study demonstrates that the cell wall-anchored PbsP protein is critical in the context of GBS infection caused by a representative strain of the hypervirulent CC17 clone. Hallmarks of CC17 strains are their tropism for the central nervous system and their specific set of adhesins. A crucial step in the pathogenesis of GBS disease is penetration of the BBB, a process dependent on the ability of these bacteria to exploit the Plg-Pln fibrinolytic system^29,34^. Such a system has a dual role in the interactions between microbial pathogens and the endothelium^39–42^. This is the case for GBS where Plg bound to endothelial cell membranes can function as a receptor for GBS adhesins, while soluble Plg can be activated to Pln, after binding to the bacterial surface, by the action of Plg activators produced by endothelial cells and other cell types. Surface-associated Pln, in turn, can degrade extracellular matrix and basement membrane proteins either directly or indirectly (e.g. through activation of matrix metalloproteases) and initiate the release of fibrinogen and bradykininogen fragments, promoting inflammation and blood vessel permeability^42–46^.

We previously showed that the CC23 strain NEM316 uses PbsP to bind human Plg, which is then activated into plasmin by tPA^34^. Moreover, PbsP-dependent Plg activation was required for hematogenous spreading of NEM316 GBS to the brain during experimental infection. In this study, we extend the role of PbsP to the virulence of the CC17 BM110 strain. First we found that, in the absence of PbsP, BM110 displayed a selective impairment in its ability to colonize the brain, despite an unchanged capacity to persist at high levels in the blood and to invade the kidney. Second, immunization with a recombinant form of the antigen largely prevented brain infection and lethality, and this also occurred by a highly selective mechanism. Accordingly, *in vitro* experiments indicated that *pbsP* deletion impaired the ability of BM110 GBS to adhere to brain endothelial cells and to transmigrate through them using plasmin-dependent mechanisms. These findings were unexpected since very low PbsP levels were originally detected on the CC17 surface^34^, in contrast to the high expression of the CC17-specific Srr2 protein, which plays a major role in the binding of both Plg and fibrinogen^30,31^.

By analyzing PbsP expression in the context of infection, we demonstrate here that *pbsP* transcription is markedly up-regulated in CC17 bacteria during mouse infection or upon contact with cultured brain endothelial cells. Our data extend those of Mereghetti and coworkers who reported profound changes in the transcriptome of the CC23 strain NEM316 when grown in the presence of human blood^47^. Strikingly, in that study, only a few virulence genes were up-regulated, and among these, the highest levels of transcription were displayed by *pbsP* (referred to as *gbs0428*) and by other genes encoding proteins implicated in binding or activation of Plg, such as enolase, glyceraldeyde 3-phosphate dehydrogenase and Skizzle. While this manuscript was under review, Cook *et al*. reported that PbsP expression is increased in CC7 GBS (strain A909) during colonization of the mouse vagina or upon contact with vaginal lavage fluid^48^. Therefore, GBS belonging to different clonal complexes selectively upregulate PbsP during interaction with host cells or host fluids, both *in vivo* and *in vitro*, suggesting a conserved role of PbsP during colonization and infection.

In contrast to our previous observations done with the CC23 strain NEM316, in which *pbsP* deletion impaired the ability to invade several organs, including the brain and the kidney, the deletion of *pbsP* in the BM110 strain impairs brain invasion only. This difference might be related to the different set of adhesins expressed at the surface of CC17 strains. For example, expression of the adhesins HgvA and Srr2 is restricted to CC17 GBS, while Srr1 is expressed by non-CC17 strains only^31^. Therefore, it is possible that CC17-specific adhesins can compensate for absence of PbsP in the context of colonization of the kidney, but not of the brain. In addition, strain-specific regulation of adhesin expression might explain this difference.

PbsP expression was shown to be repressed by the CovRS TCS^49–51^ in CC23 GBS^35^, but we observe in this study that CovRS-dependent regulation is not conserved in the BM110 strain. Instead, Cook *et al*. recently reported that PbsP expression is directly regulated by the SaeRS two-component system in CC7 GBS^48^. Therefore, further studies will be needed to analyze the role of SaeRS and other regulation systems in controlling PbsP expression in GBS strains belonging to different clonal complexes and the external environment signal triggering *in vivo* PbsP expression.

In conclusion, our studies indicate that PbsP is a conserved Plg binding adhesin, is expressed during infection, and is necessary for brain invasion by GBS belonging to the major CC responsible for neonatal meningitis. As such, this protein might represent a promising candidate for inclusion in a universal GBS vaccine.

## Materials and Methods

### Bacterial strains and reagents

The GBS strain BM110, a human clinical isolate and a prototype strain belonging to the hypervirulent CC17 clonal complex^7^, was used throughout this study. The BM110 Δ*pbsP* and BM110 Δ*covR* GBS mutants were obtained as previously described, using pG1 shuttle plasmids containing gene-specific deletion cassettes^10,34^. A Δ*pbsP* strain carrying a complementing vector with constitutive *pbsP* expression (Δ*pbsP* + *pbsP*) was obtained as previously described^34^. Recombinant PbsP was produced as a fusion with glutathione-S-transferase (GST) from *Escherichia coli* BL21(DE3) after transformation with the pGEX-SN_PbsP plasmid, and purified as previously described^34,52^. Mouse anti PbsP sera were raised in CD1 mice (5 weeks old, Charles River Labs) as previously described^34^. Briefly, the mice were bled at 2 weeks after the last immunization and the sera were tested for reactivity to the purified antigen using ELISA^15,52^.

### Cell wall extracts and immunoblots

Cell wall extracts were obtained by mutanolysin extraction in a hypertonic sucrose buffer and used for Western blot analyses as described previously^52,53^. Briefly, 10 µg of cell wall proteins were run on polyacrylamide gels (SDS-PAGE), transferred to a nitrocellulose membrane and PbsP was visualized by chemi-luminescence after incubation with mouse anti-PbsP serum and horseradish peroxidase conjugated goat anti mouse IgG (Abcam ab6789).

### Mouse meningitis model

The CC17 BM110 strain and the deletion mutants were used to induce infection in 8-week-old CD1 female mice, as previously described^34,52^. Mice were infected i.v with 1 × 10^8^ CFU and monitored every 12 hours for clinical signs and lethality. Animals with signs of sepsis or neurological manifestations were humanely euthanized and GBS invasion of organs confirmed as the cause of disease, as described^34^. In a second set of experiments GBS-infected mice were sacrificed at 48 h after infection to collect blood, brains and kidneys. The number of CFU was measured in organ homogenates using standard methods, as described^53^. The levels of MPO, IL-1β and TNF-α were measured in brain homogenates by using commercially available kits purchased from R&D, as previously described^54^. The protective effect of PbsP immunization was evaluated as previously described^34^. Briefly, CD1 female mice were immunized by intraperitoneal injection with 30 µg of recombinant PbsP or GST in complete (first injection) or incomplete (second and third injection) Freund’s adjuvant emulsions (in a total volume of 0.2 ml) on day 0, 14 and 28. Three week after the last immunization, mice were challenged i.v. with 1 × 10^8^ CFUs of BM110 WT bacteria. All studies involving mice were performed in accordance with the European Union guidelines for the use of laboratory animals. The procedures were approved by the animal welfare committee of University of Messina (OPBA permit n. 18052010) and by the Ministero della Salute of Italy (permit n° 665/2015). Data were analyzed using GraphPad Prism 5.0 (GraphPad Software, San Diego, California).

### Quantitative RT-PCR

To measure *pbsP* mRNA, total RNA was extracted from the organs of infected mice or from bacteria exposed *in vitro* to different conditions, retro-transcribed and analyzed by RT-PCR. The first step in isolation of bacteria from infected brains or kidneys (obtained from CD1 mice at 24 h after i.v. challenge with 1 × 10^8^ CFUs of BM110 WT bacteria) consisted in homogenizing the organs with the gentle MACS Dissociator system (Mylteni), according to manufacturer’s instructions. Next, tissue debris and residual intact cells were sedimented by centrifugation at 200 g for 10 min and bacteria were recovered from supernatants by high speed centrifugation (12,000 g for 10 min). In *in vitro* experiments, 5 × 10^6^ GBS grown to the exponential phase in TH broth were added to 0.5 ml of brain microvascular endothelial cell cultures or to fresh, cell-free, medium in 24 well plates. After centrifugation at 500 g for 2 min, the plates were incubated at 37 °C for 2 h. After scraping the contents of the wells were collected and bacteria were recovered by differential centrifugation, as described above.

To extract RNA, bacterial pellets were suspended in 350 µl of Tris-HCl (pH 8, 10 mM) in 1.5 ml microcentrifuge tubes, to which 25 µg of glass beads (106 µm, Sigma-Aldrich) were added. The tubes were placed in a RETSH MM30 homogenizer and shaken at 30 Hz for 20 minutes. RNA in the homogenized samples was purified using Qiagen RNA purification columns and stored at −80 °C after quantification by Nanodrop 2000 (Thermo Fischer) readings and gel electrophoresis analysis. Reverse transcription was performed with the M-MLV reverse transcriptase (Invitrogen) and random primers (Promega). Specific primer pairs were designed to obtain amplicons of 68 (*pbsP*) and 74 bp (*gyrA*), as shown in Table S1, Supplementary Methods. Quantitative PCR (qPCR) was carried out with the Taqman Gene Expression Master MIX (Applied Biosystem) in a 7500 Real-Time PCR Detection System (Applied Biosystem). Relative gene expression levels were calculated with the ΔΔCT method^55,56^ where expression values were normalized with the expression of the housekeeping *gyrA* gene. Each assay was performed in triplicate. Expression of the PbsP protein on the bacterial surface was analyzed, in selected experiments, by immuno-fluorescence flow cytometry using anti-PbsP mouse sera, as described^34^.

### Adhesion, invasion and transmigration assays

The human brain endothelial cell line hCMEC/D3 was provided by P.O. Couraud (INSERM, Paris, France) and the adherence and invasion assays were performed as described^15,57^. Briefly, bacteria were grown to the mid-log phase and added to confluent monolayers at a multiplicity of infection (MOI) of 20 bacteria/cell. After a one-hour incubation, monolayers were washed with PBS to remove non-adherent bacteria, lysed, and plated to enumerate cell-associated bacteria. For the invasion assay, after washing, the monolayers were further incubated for 1 h with medium supplemented with penicillin and streptomycin (200 units/ml 100 µg/ml, respectively) to kill extracellular bacteria. Percentages of bacterial adhesion and invasion were calculated as 100× (recovered cfu/initial inoculum cfu). To test the transmigration ability of BM110, an endothelial blood-brain barrier *in vitro* model was used by cultivating hCMEC/D3 cells in collagen coated-polycarbonate transwell membrane inserts with a pore size of 3 µm, as previously described^15,57^. This model allows access to the upper (“blood” side) and lower (“brain side”) chambers and mimics GBS penetration into the brain. The hCMEC/D3 cells were grown for 5–7 days at 37 °C in a humidified chamber containing 5% CO_2_ to reach confluence. Prior to the assay, the integrity of the monolayer was verified by adding the Evans blue stain to the upper chamber. The hCMEC/D3 cells were then washed and resuspended in serum-free culture medium without antibiotics. Log-phase GBS were added to the upper chamber together with Plg and/or tPA. At 2 h post-infection, the lower chamber medium was entirely removed and plated onto TH agar to measure transmigration.

## Electronic supplementary material

Supplementary Information
