# Supplementary material for: The plasminogen binding protein PbsP is required for brain invasion by hypervirulent CC17 Group B streptococci
Source: Sci Rep. 2018 Sep 25;8:14322. doi: 10.1038/s41598-018-32774-8 (PMC6156580; doi:10.1038/s41598-018-32774-8)
Supplement: Supplementary file 1 — Supplementary Information [file 41598_2018_32774_MOESM1_ESM.pdf]

The plasminogen binding protein PbsP is required for brain invasion by hypervirulent CC17 Group B streptococci

Germana Lentini[1]*, Angelina Midiri[1]*, Arnaud Firon[2], Roberta Galbo[3], Giuseppe Mancuso[1], Carmelo Biondo[1], Emanuela Mazzon[4], Annamaria Passantino[5], Letizia Romeo[4], Patrick Trieu-Cuot[2], Giuseppe Teti[1,6§] and Concetta Beninati[1,7]

[1]*Metchnikoff Laboratory, Departments of Human Pathology and Medicine, University of Messina, Messina, Italy;* [2]*Institut Pasteur, Unité de Biologie des BactériesPathogènes à Gram Positif, CNRS ERL3526, 75015 Paris, France;* [3]*Department of Chemical, Biological, Pharmaceutical and Environmental Sciences, University of Messina, Messina, Italy;*[4]*IRCCS Centro Neurolesi Bonino Pulejo, Messina,*[5]*Department of Veterinary Sciences, University of Messina, Messina, Italy;* [6]*Charybdis Vaccines Srl, Messina, Italy;* [7]*Scylla Biotech Srl, Messina, Italy*

Figure-S1 (Teti)

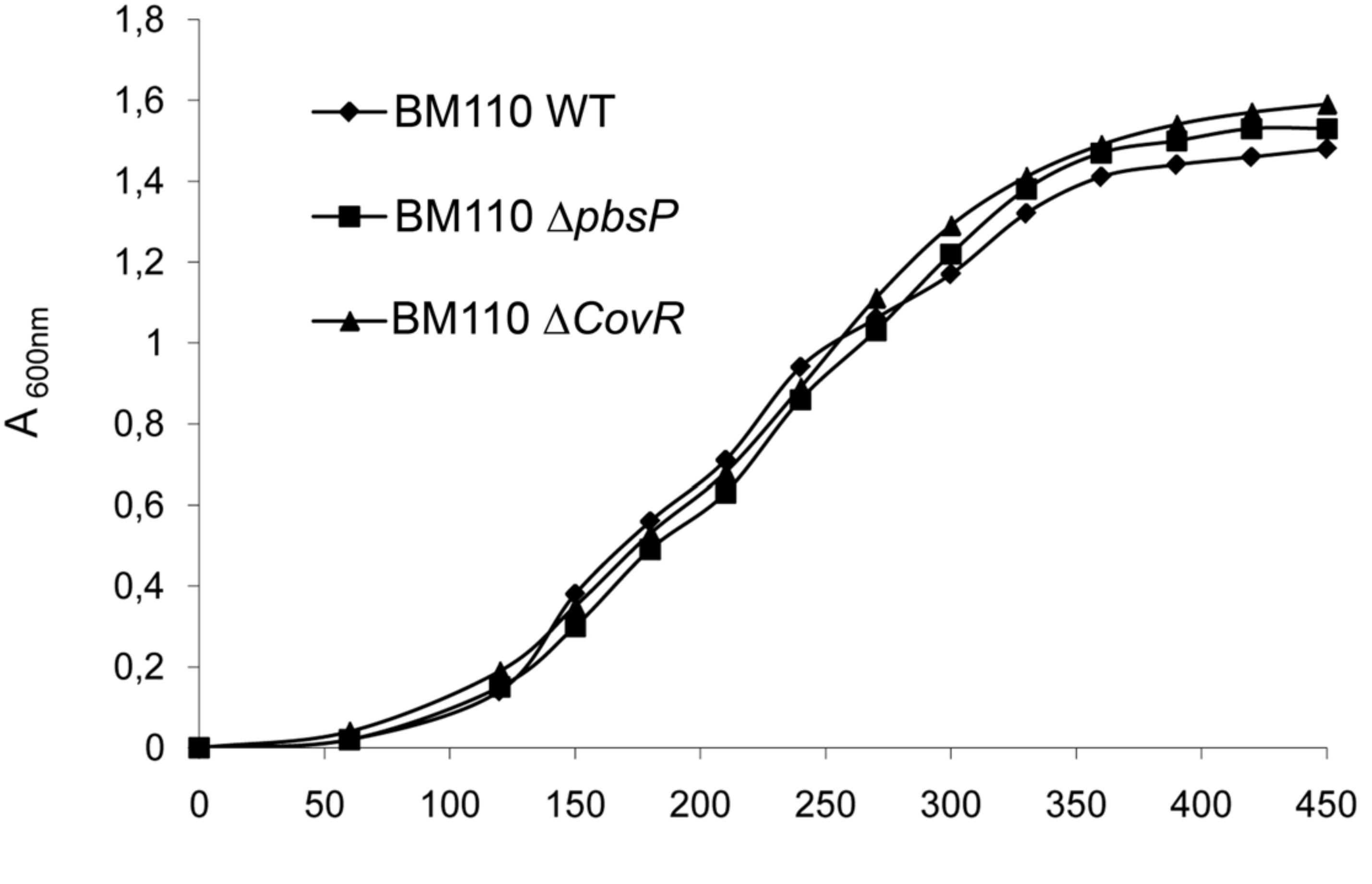

Fig. S2 (Teti)

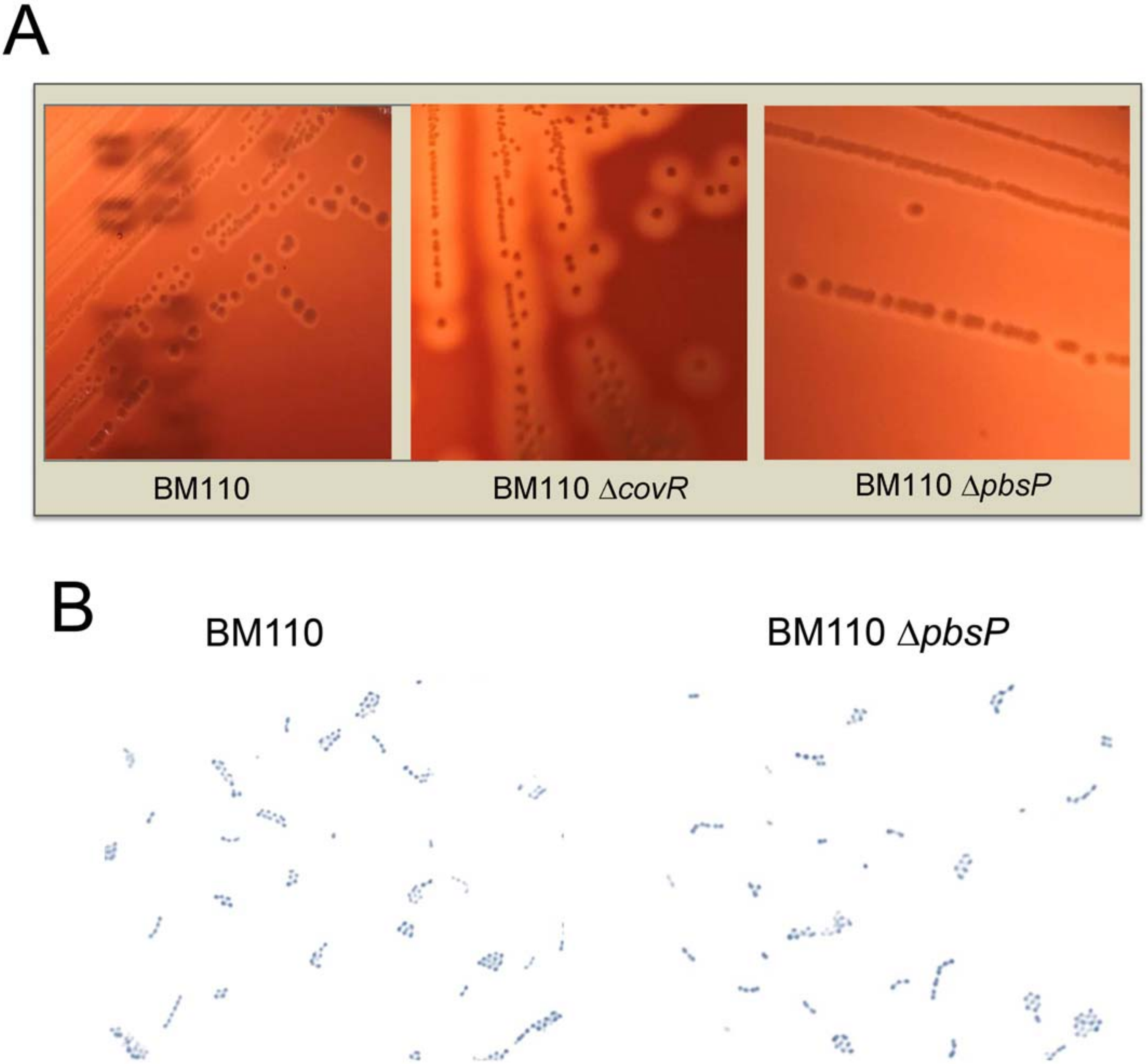

Fig. S3 (Teti)

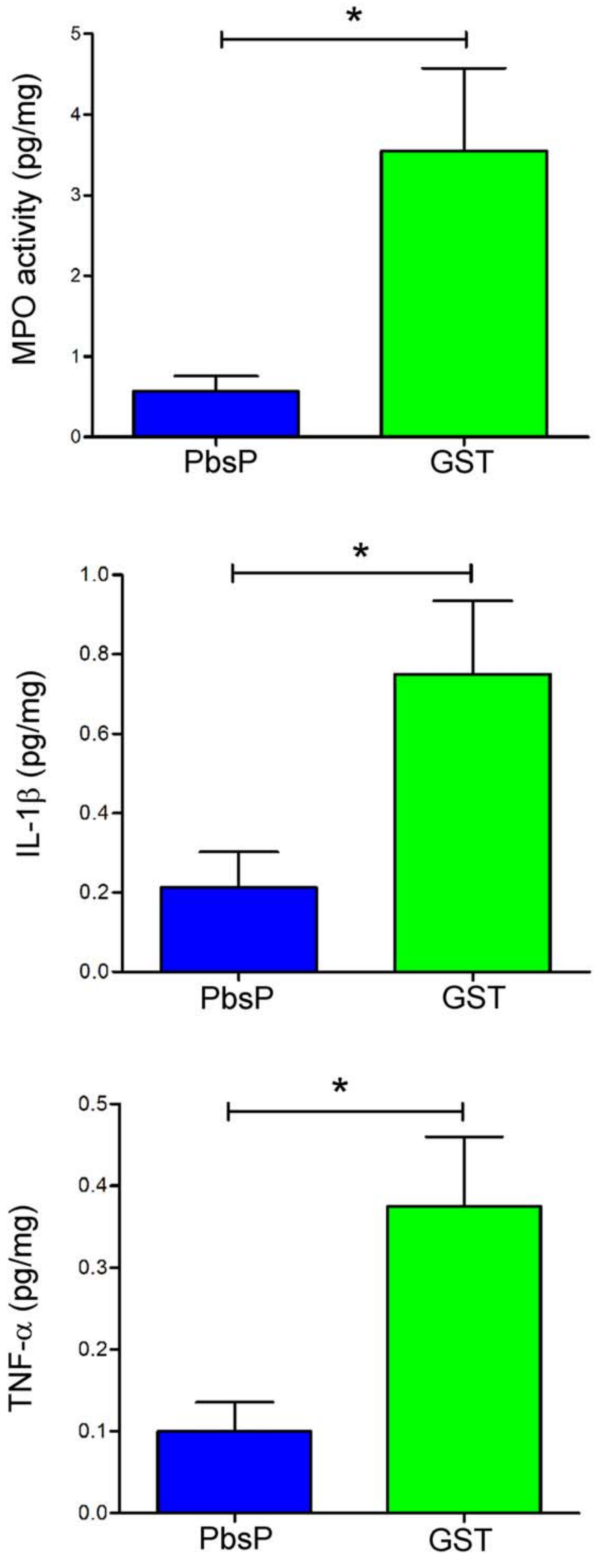
*
MPO activity (pg/mg)
0
1
2
3
4
5
PbsP
GST
*
IL-1β (pg/mg)
0.0
0.2
0.4
0.6
0.8
1.0
PbsP
GST
*
TNF-α (pg/mg)
0.0
0.1
0.2
0.3
0.4
0.5
PbsP
GST

Figure-S4 (Teti)

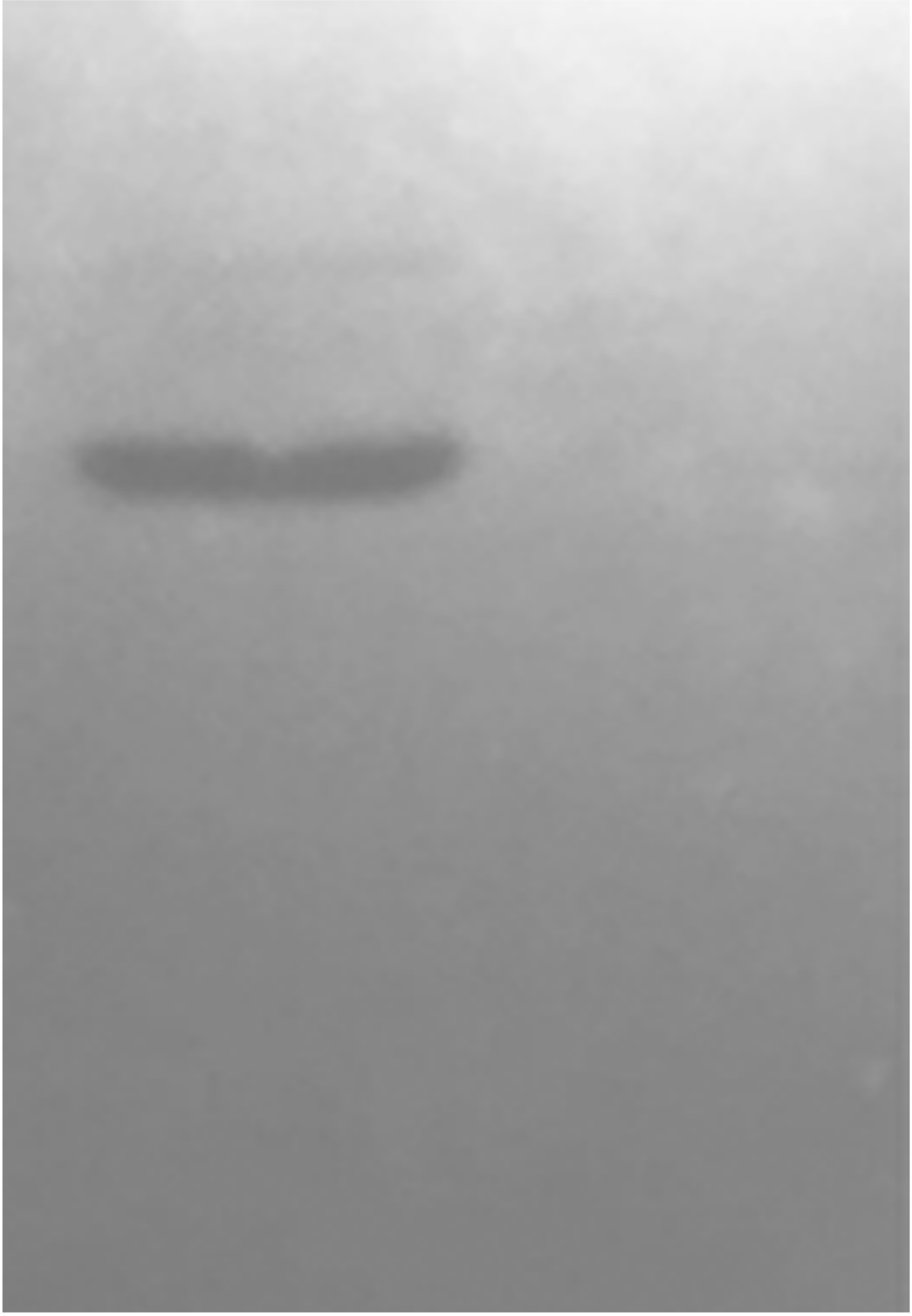

Figure S1. Growth in Todd-Hewitt broth of GBS BM110 mutants.

Growth curves of BM110 (WT, diamonds), *pbsP* deletion mutant (BM110 Δ*pbsP,* squares*)* and *CovR* deletion mutant (BM110 Δ*CovR,* triangles) GBS strains in Todd-Hewitt broth.

Fig. S2. Colony morphology and microscopic appearance of GBS BM110 mutants.

(A) Colony morphology of wild-type BM110 strain (BM110) and its *CovR* or *pbsP* deletion mutants (BM110 Δ*CovR* or BM110 Δ*pbsP*, respectively*)* on blood agar. **(B)** Cell morphology after Gram stain of wild-type BM110 strain (BM110) and its *pbsP* deletion mutant (BM110 Δ*pbsP*).

Fig. S3. PbsP-based immunization prevents GBS-induced encephalitis. MPO, IL-1β and TNF-α protein levels were measured in organ homogenates from mice immunized with recombinant PbsP fused to glutathione-S-transferase (GST-PbsP) or with GST as a control. Data are expressed as means ± standard deviations of three duplicate observations, each conducted with a different animal, in the course of a single experiment. *, $p<0.05$ by one way ANOVA.

Figure S4. Image from the same blot shown in Fig 1, obtained with a higher exposure

#

Table S1

RT-PCR primer and probe sequences

pbsp_up	TGATGGTGGCTACCTCTGTTAGAA
pbsp_low	AGTCGTTTTGGTCATCGCTACA
gyr_up	AACTGGGAAGGGATCAATTGTCT
gyr_low	CTTTCCCATTTGAAGTTGTTTCAA
FAM-pbsp_probe	ATGGCAACGCCGACAC
VIC-gyr_probe	ACGTTCACGGACTGAA
